# Transcriptome and Metabolome Analyses Reflect the Molecular Mechanism of Drought Tolerance in Sweet Potato

**DOI:** 10.3390/plants13030351

**Published:** 2024-01-24

**Authors:** Yumeng Yin, Shouchen Qiao, Zhihe Kang, Feng Luo, Qianqian Bian, Guozheng Cao, Guorui Zhao, Zhihao Wu, Guohong Yang, Yannan Wang, Yufeng Yang

**Affiliations:** 1 Cereal Crop Research Institute, Henan Academy of Agricultural Sciences, Postgraduate T&R Base of Zhengzhou University, Zhengzhou 450002, China; yinyumeng0521@163.com; 2 School of Agricultural Sciences, Zhengzhou University, Zhengzhou 450001, China; 3 Cereal Crop Research Institute, Henan Academy of Agricultural Sciences, Zhengzhou 450002, China; 13676963200@163.com (S.Q.); kangzhihe@163.com (Z.K.); bianqianqian11@163.com (Q.B.); 19939709506@163.com (G.C.); zhaoguorui2032@163.com (G.Z.); 15738517757@163.com (Z.W.); yangguohong9211@163.com (G.Y.); 4 Henan Provincial Center of Seed Industry Development, Zhengzhou 450007, China; hnpzdj@163.com

**Keywords:** sweet potato, drought, transcriptome, metabolome, respiration metabolism, antioxidant system

## Abstract

Sweet potato (*Ipomoea batatas* (L.) Lam.) is one of the most widely cultivated crops in the world, with outstanding stress tolerance, but drought stress can lead to a significant decrease in its yield. To reveal the response mechanism of sweet potato to drought stress, an integrated physiological, transcriptome and metabolome investigations were conducted in the leaves of two sweet potato varieties, drought-tolerant zhenghong23 (Z23) and a more sensitive variety, jinong432 (J432). The results for the physiological indexes of drought showed that the peroxidase (POD) and superoxide dismutase (SOD) activities of Z23 were 3.68 and 1.21 times higher than those of J432 under severe drought, while Z23 had a higher antioxidant capacity. Transcriptome and metabolome analysis showed the importance of the amino acid metabolism, respiratory metabolism, and antioxidant systems in drought tolerance. In Z23, amino acids such as asparagine participated in energy production during drought by providing substrates for the citrate cycle (TCA cycle) and glycolysis (EMP). A stronger respiratory metabolism ability could better maintain the energy supply level under drought stress. Drought stress also activated the expression of the genes encoding to antioxidant enzymes and the biosynthesis of flavonoids such as rutin, resulting in improved tolerance to drought. This study provides new insights into the molecular mechanisms of drought tolerance in sweet potato.

## 1. Introduction

Plants are exposed to various environmental stresses during growth and development. Among these, drought is one of the most serious environmental pressures affecting plant productivity, food production safety, and sustainable agricultural development [1,2]. Sweet potato (*Ipomoea batatas* (L.) Lam.) ranks eighth in the world in terms of production, and China is the largest producer of sweet potato [3]. Sweet potato has extensive adaptability to various environmental conditions, with high yield and a strong stress tolerance [4]. Thus, it can be planted on barren, marginal land and is often used as an ideal crop in disaster years. Despite these qualities, drought is still a major threat limiting the growth and yield of sweet potato, and it is of great importance to study the plant’s drought tolerance mechanisms with the aim of developing new cultivars to counter drought stress [5,6].

Plant response to drought stress is a complex biological process, involving stress perception, signal transduction, gene expression regulation, and various physiological processes. To date, many molecular pathways related to drought tolerance have been identified. After perceiving drought stress, reactive oxygen species (ROS) are generated in plants, and various stress signaling pathways (e.g., mitogen-activated protein kinase; Ca^2+^; plant hormone) are simultaneously stimulated to regulate downstream gene expression and plant physiological responses [7]. Plant hormones, such as abscisic acid (ABA), jasmonic acid (JA), and ethylene (ET), are key signal regulators in many vital processes. The cooperated regulation of ABA and ET signaling in response to drought stress is necessary for the fine-tuning of stomatal opening and closure, thereby limiting transpirational water loss and adjusting the drought resistance of plants [8]. JA can interact with hormones such as gibberellinic acid (GA), salicylic acid (SA), ET, and ABA to regulate environmental responses [9]. ROS are among the earliest and most common stress signal molecules produced in response to drought stress, and are a key signal hub for integrating different stress signal networks [10,11,12]. ROS and antioxidants exert many of their effects through the redox-dependent regulation of components of hormone signaling [13]. Moreover, ROS signaling can be linked to calcium and phosphorylation signaling through the functions of respiratory burst oxidase homologues, aquaporins, Ca^2+^ channels, and various kinases and phosphatases, subsequently triggering transcriptional responses to stress [14]. Specific TFs are upregulated or downregulated by protein kinases or phosphatases, and they bind to the *cis*-elements of corresponding stress genes, suppressing or enhancing their transcription [15]. This regulatory role of TFs can impact the plant’s response to different stress conditions, leading to various stress responses. Many transcription factor families, such as MYB, bHLH, bZIP, WRKY, and NAC, have been characterized, which play important roles in enhancing plant drought tolerance [16]. For example, as a typical antioxidant, anthocyanins are closely associated with the drought resistance of plants, and their content is regulated by TFs and structural genes. The MYB, bHLH, and WD40 TFs can individually regulate the structural genes in the anthocyanin biosynthetic pathway, or form the conserved MBW complex to collectively regulate these genes [17,18]. ABA plays a crucial role in the drought-stress responses of higher plants. Many drought-related gene-promoter regions contain conserved ABA-responsive elements (ABRE). The AREB/ABF transcription factors (TFs), which are basic leucine zipper (bZIP)-type TFs capable of binding to ABRE, can activate the expression of ABA-dependent genes under drought stress. The ABA-dependent pathway is one of the primary pathways for responding to drought stress [19,20].

Although ROS are key signals that enable cells to respond quickly to drought stress, excessive ROS can affect cell membrane properties, cause damage to nucleic acids, and affect the expression of many genes controlling biological processes [21]. To protect plants from oxidative damage, plant cells and their organelles employ antioxidant defense systems. The components of the antioxidant defense system are enzymatic antioxidants (e.g., SOD, POD, catalase (CAT)), and non-enzymatic antioxidants (e.g., glutathione, flavonoids, and amino acids) [22]. SOD has been proposed to be important in plant stress tolerance and provides the first line of defense against the toxic effects of elevated levels of ROS. SOD can convert harmful superoxide radicals into hydrogen peroxide. Then, through the action of CAT and POD, they are decomposed into completely harmless water. Flavonoids and other non-enzymatic antioxidants serve as ROS scavengers by locating and neutralizing radicals before they damage the cell [21]. Furthermore, drought stress can lead to a decreased respiration rate, resulting in blocked electron transport and reduced energy production [23]. As respiration is pivotal for plant energy production, exploring changes in gene expression and metabolite accumulation in respiration during drought stress will aid in a better understanding of how plants cope with this adversity.

With the rapid development of bioinformatics, increasingly advanced sequencing technologies are employed to study the mechanisms of plant stress tolerance, including transcriptomics and metabolomics. The metabolome is a bridge connecting the changes between plant transcriptomes and phenotypes. Integrated analyses of transcriptomes and metabolomes are now widely used to identify key differences in regulatory mechanisms between stress-tolerant and -sensitive genotypes displaying differential phenotypes, and relevant studies have shown that plants respond to drought stress mainly by regulating oxidative balance, signal transduction, plant hormones and energy metabolism [24,25,26,27,28]. In sweet potato, transcriptomics has become a powerful tool for examining various mechanisms, such as potassium-deficiency tolerance [29], skin color [30], and stem nematode resistance [31]. In this study, we used integrated transcriptome–metabolome analyses to reveal the molecular differences between drought-sensitive and drought-tolerant sweet potato cultivars under drought stress, providing further insights into the drought tolerance mechanisms of this important crop.

## 2. Results

### 2.1. Morphological and Physiological Responses under Drought Stress

**Figure 1:**
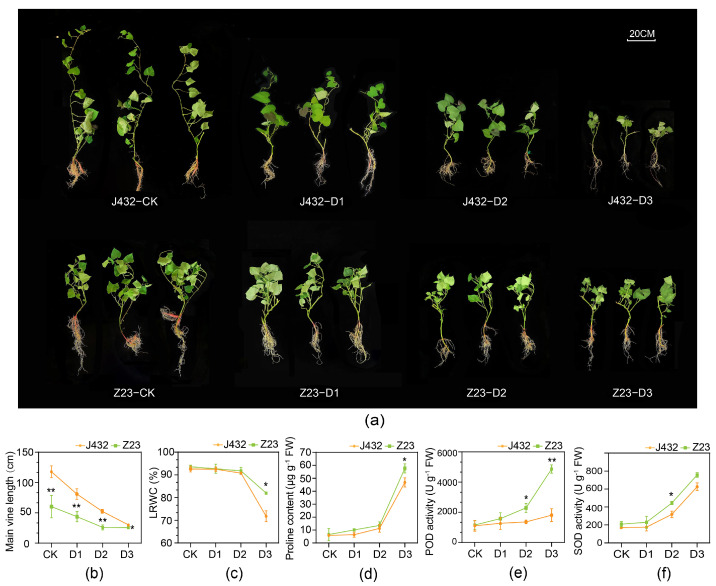
Morphological traits and physiological indices of J432 and Z23 at different drought levels. (**a**) Morphological traits of J432 and Z23 after the conclusion of the entire drought treatment. (**b**) Main vine length. (**c**) Leaf’s relative water content. (**d**) Proline content. (**e**) POD activity. (**f**) SOD activity. * and ** indicate significant differences (at *p* < 0.05 and *p* < 0.01, respectively) between J432 and Z23 at each drought level based on Student’s *t*-test.

To study the mechanisms of sweet potato response to drought stress, drought-sensitive cultivar J432 and drought-tolerant cultivar Z23 were subjected to natural drought treatment at four relative soil moisture content (RSMC) levels, namely, CK (45% RSMC), D1 (25% RSMC as mild drought stress), D2 (15% RSMC as moderate drought stress) and D3 (6% RSMC as severe drought stress). With increasing degrees of drought, the growth of J432 and Z23 gradually became inhibited (Figure 1a). After three weeks’ treatment, the main vine length of Z23 at D3 decreased by 57.47% compared with that at CK, while J432 decreased by 74.60%, indicating that J432 was more affected by drought stress than Z23 (Figure 1b). At CK level, the leaf relative water content (LRWC) of Z23 was 93.55%, and that of J432 was 92.57%. By contrast, the LRWC of Z23 decreased to 82.7% at the D3 level while J432 decreased further, to 75.1% (Figure 1c). This result showed that the leaves of Z23 had a stronger water-retention capacity, which enabled Z23 to better maintain its growth and metabolic activities compared to J432 under water-deficit conditions. There was no significant difference in proline content between J432 and Z23 at the CK level. However, proline accumulated dramatically in both cultivars with an increasing degree of drought. At the D3 level, the proline content of Z23 (57.63 μg g^−1^ FW) was 23.17%, which was significantly greater than that of J432 (46.79 μg g^−1^ FW) (Figure 1d). Both SOD and POD activities in J432 and Z23 exhibited a rising trend from CK to D3, and were higher in Z23 than in J432 at each drought level. At the D3 level, the POD activity of Z23 was 169.47% higher than that of J432, and the SOD activity was 21.11% higher than that of J432 (Figure 1e,f).

### 2.2. Transcriptome Analysis of Sweet Potato under Drought Stress

To identify differentially expressed genes (DEGs) involved in drought stress in sweet potato leaves, RNA-seq was performed. The raw reads for each sample ranged from 38.41 to 49.20 million. After removing low-quality reads, clean reads ranged between 37.13 and 48.09 million. The Q20 and Q30 values were greater than 96.60% and 91.05%, respectively. Approximately 79.77–83.59% of clean reads were mapped to the sweet potato wild relative *Ipomoea trifida* genome (Table S1). The transcriptome data of the twenty-four samples described in the study were deposited into the National Center for Biotechnology Information (NCBI) databases, and the bioproject accession number is PRJNA1039563.

**Figure 2:**
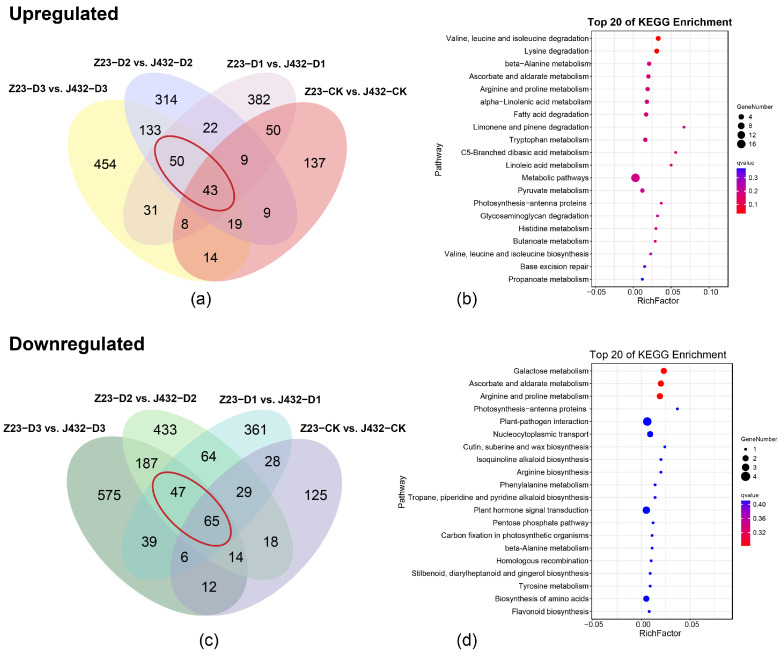
Transcriptome analysis. (**a**) Venn diagram of upregulated DEGs in Z23 compared with J432 at the CK, D1, D2, and D3 levels. The red circles are 93 upregulated DEGs in Z23 compared with J432 at the D1, D2, and D3 levels. (**b**) KEGG pathway analysis of 93 common DEGs in panel (**a**). (**c**) Venn diagram of downregulated DEGs in Z23 compared with J432 at the CK, D1, D2, and D3 levels. The red circles are 112 downregulated DEGs in Z23 compared with J432 at the D1, D2, and D3 levels. (**d**) KEGG pathway analysis of 112 common DEGs in panel (**c**).

Principal component analysis (PCA) showed that the repeatability in each group was good and the treatment groups were clearly separated from each other (Figure S1a). In J432, a total of 939 DEGs were identified in D1 vs. CK (370 upregulated and 569 downregulated), 4182 in D2 vs. CK (1914 upregulated and 2268 downregulated), and 11,055 in D3 vs. CK (5092 upregulated and 5963 downregulated). In Z23, a total of 331 DEGs (179 upregulated and 152 downregulated) were identified in D1 vs. CK, 5156 in D2 vs. CK (2194 upregulated and 2962 downregulated), and 9238 in D3 vs. CK (4207 upregulated and 5031 downregulated) (Figure S1b). a Kyoto Encyclopedia of Genes and Genomes (KEGG) and Gene Ontology (GO) analysis was conducted on the 93 upregulated DEGs and 112 downregulated DEGs in Z23 compared with J432 at the D1, D2, and D3 levels (Figure 2a–c, Table S2). According to the KEGG annotations, 93 upregulated DEGs were majorly related to the ascorbate and aldarate metabolism, alpha-linolenic acid metabolism, pyruvate metabolism, and amino acids metabolism (Figure 2b). A total of 112 downregulated DEGs were strongly related to ascorbate and aldarate metabolism, phenylalanine metabolism, flavonoid biosynthesis and amino acids metabolism (Figure 2d). According to GO analysis, both groups of DEGs were significantly enriched in biological processes. A total of 93 upregulated DEGs were mainly enriched in cell-growth-related terms, such as unidimensional cell growth, developmental cell growth, and cell tip growth (Figure S1c). A total of 112 downregulated DEGs were mainly enriched in transport-related terms such as the regulation of transport, regulation of ion transport, regulation of metal ion transport (Figure S1d).

We observed that differentially expressed transcripts encoded TFs between Z23-D1 vs. J432-D1, Z23-D2 vs. J432-D2 and Z23-D3 vs. J432-D3 groups (Table S3). One hundred and fifty-eight TFs were categorized into various families, including ERFs, bHLHs, GRASs, MYB-related, and WRKYs (Figure S2a). According to KEGG annotation, these TFs were strongly related to plant hormone signal transduction and MAPK signaling pathway–plant relationships (Figure S2b).

### 2.3. Weighted Gene Co-Expression Network Analysis

**Figure 3:**
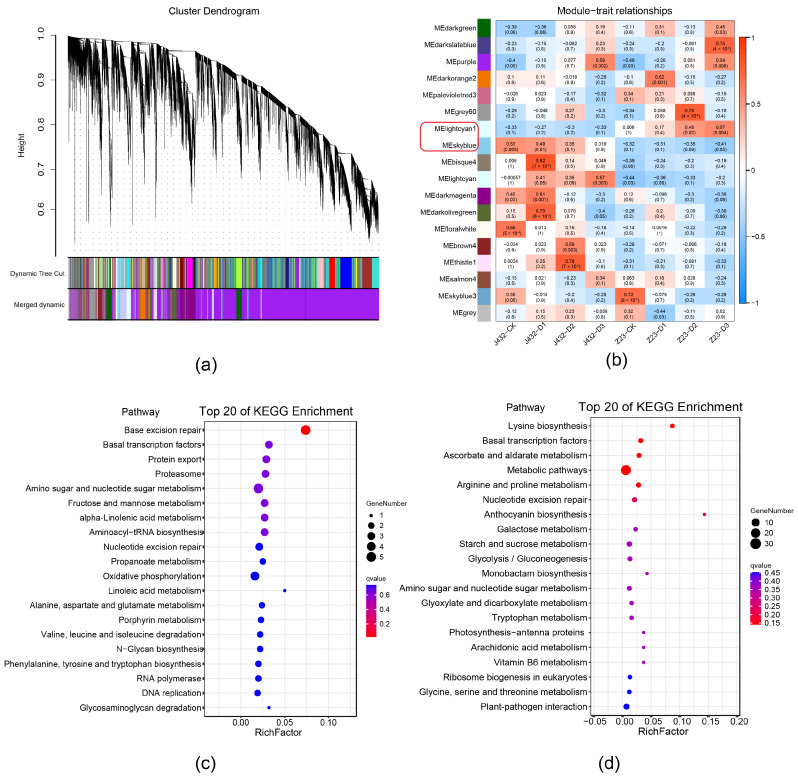
Weighted gene co-expression network analysis. (**a**) Hierarchical clustering tree showing 18 co-expressed gene modules. (**b**) Relationship among co-expression modules and samples. Blue color indicates negative correlations and red color indicates positive correlations. *p*-values are shown inside parentheses. The ‘lightcyan1’ and ‘skyblue’ modules in the red frame circle were selected for further KEGG analysis. (**c**) KEGG pathway analysis of DEGs in ‘lightcyan1’ module. (**d**) KEGG pathway analysis of DEGs in ‘skyblue’ module.

To further examine the drought-tolerance mechanisms of sweet potato, all DEGs in Figure S1b were analyzed by weighted gene co-expression (WGCNA), and a total of 18 modules (referred to by their software-assigned color) were generated (Figure 3a,b). In the ‘lightcyan1’ module, the expression levels of most DEGs increased as drought stress became more severe in Z23, and were higher for Z23 than for J432 at each drought treatment level. The DEGs expression level of the ‘skyblue’ module was opposite to that of ‘lightcyan1’ (Figure S3). The expression profiles of the DEGs in these two modules indicated that they were highly related to the stronger drought tolerance of Z23 compared with J432. Therefore, the ‘lightcyan1’ and ‘skyblue’ modules were selected for further KEGG analyses. KEGG enrichment showed that ‘lightcyan1’ module significantly enriched genetic information processing and metabolism pathways. In genetic information processing, base excision repair, basal transcription factors, and protein export pathways were significantly enriched. In metabolism pathways, many DEGs were annotated with amino sugar and nucleotide sugar metabolism, fructose and mannose metabolism, and oxidative phosphorylation (OXPHOS) (Figure 3c). The ‘skyblue’ module is significantly enriched in metabolism pathways such as the lysine biosynthesis, ascorbate and aldarate metabolism, arginine and proline metabolism, anthocyanin biosynthesis, atarch and sucrose metabolism, and EMP. In addition, basal transcription factors were, again, significantly enriched (Figure 3d).

### 2.4. Quantitative Real-Time PCR Analysis

**Figure 4:**
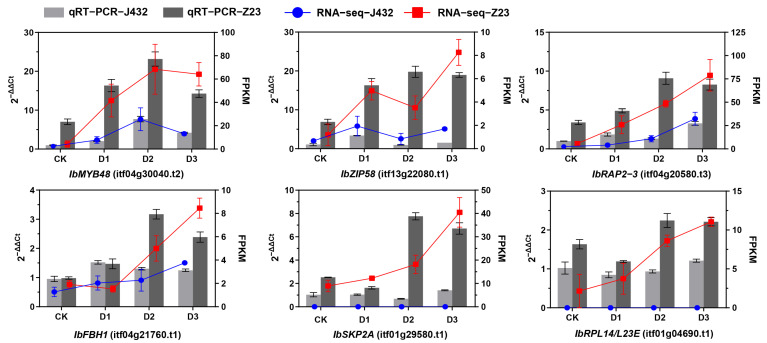
qRT-PCR verification of six genes. *IbMYB48*, myb domain protein; *IbZIP58*, basic leucine-zipper; *IbRAP2–3*, ethylene-responsive element binding protein; *IbFBH1*, basic helix–loop–helix (bHLH) DNA-binding superfamily protein; *IbSKP2A*, F-box/RNI-like superfamily protein; *IbRPL14/L23E*, ribosomal protein, L14p/L23e family protein.

Six DEGs were selected for quantitative real-time PCR (qRT-PCR) verification from the DEGs whose expression in Z23 was more than twofold higher than that of J432 during the D2 and D3 periods. Among these genes, *IbMYB48* (itf04g30040.t2), *IbZIP58* (itf13g22080.t1), *IbRAP2–3* (itf04g20580.t3), and *IbFBH1* (itf04g21760.t1) are TFs; *IbSKP2A* (itf01g29580.t1) is an F-box/RNI-like superfamily protein; and *IbRPL14/L23E* (itf01g04690.t1) is a ribosomal protein. The expression profiles of these six genes were basically consistent with the fragments per kilobase of transcript per million mapped reads (FPKM) values of the RNA-seq data, verifying the reliability of the transcriptome data (Figure 4).

### 2.5. Metabolome Analysis of Sweet Potato under Drought Stress

**Figure 5:**
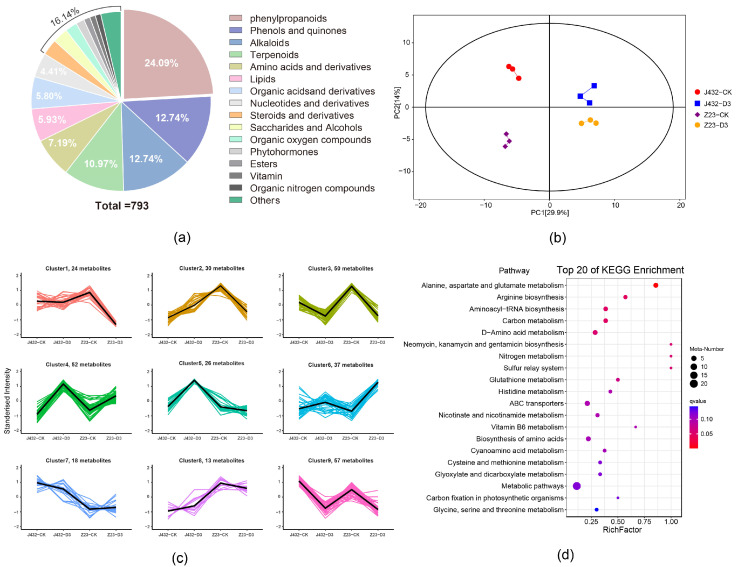
Widely targeted metabolome analysis of J432 and Z23 metabolic profiles under control (CK) and severe drought (D3) conditions. (**a**) All DAM classifications. (**b**) PCA of DAMs. (**c**) K-means analysis of DAMs. The thick black line represents the mean value of gene expression patterns in the class, each line in the figure represents a metabolite, and different classes are shown with different colored lines. (**d**) KEGG pathway analysis of 61 DAMs in clusters 1 and 6 of panel (**c**).

In this study, widely targeted metabolomics was used to detect and analyze the metabolites in J432 and Z23 samples. At the CK and D3 levels, 793 metabolites were identified in J432 and Z23, including 73 negative ion-mode metabolites and 720 positive ion-mode metabolites (Table S4). Phenylpropanoids were the most common among all the identified metabolites, representing 24.09% of the total. Among these phenylpropanoids, flavonoids were the most abundant, constituting 60.21% of all phenylpropanoids (Figure 5a). PCA showed that the repeatability in each group was good, and the groups were significantly different from each other (Figure 5b). Compared with CK, 170 differentially accumulated metabolites (DAMs) (90 upregulated and 80 downregulated) were identified in J432 at D3, and 186 DAMs (66 upregulated and 120 downregulated) were identified in Z23 at D3. According to the accumulation patterns, DAMs were divided into 10 groups by K-means analysis (Figure 5c).

The expression trends of DAMs in clusters 1, 3, 4, 6, and 9 were consistent in J432 and Z23. Among these clusters, the content changes in DAMs in clusters 1 and 6 were more pronounced in Z23 compared with J432. Therefore, 61 DAMs from clusters 1 and 6 were selected for further KEGG enrichment analysis (Table S5). The results indicated that DAMs were enriched in the metabolism for several amino acids, glutathione metabolism, ABC transporters, and several other pathways (Figure 5d). Additionally, a total of 17 out of 61 DAMs were phenylpropanoids, and these 17 phenylpropanoids included nine flavonoids, such as saponins, pinocembrin, and puerarin. Except for fisetin, the accumulation levels of flavonoids were upregulated under drought stress.

### 2.6. Integrated Transcriptome–Metabolome Analysis

**Figure 6:**
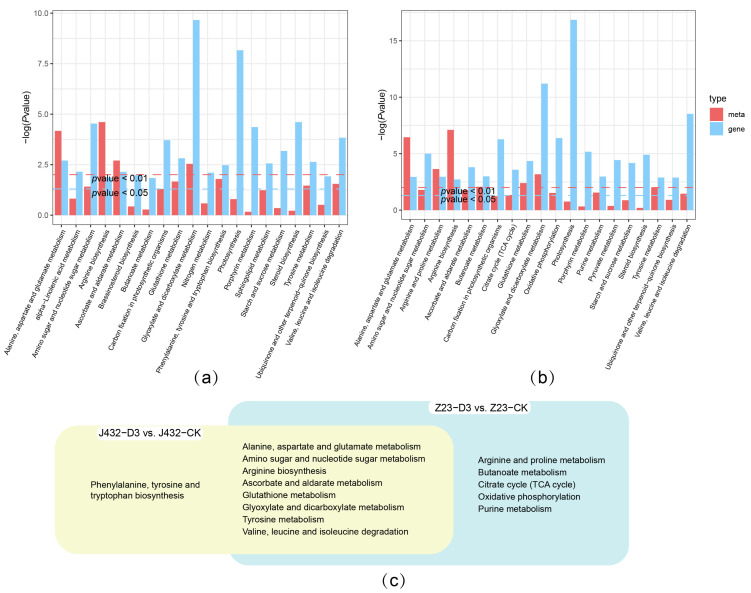
Integrated transcriptome–metabolome analysis of J432 and Z23 under control (CK) and severe drought (D3) conditions. (**a**) Transcriptome and metabolome combined KEGG analysis of J432 at D3 vs. CK group. (**b**) Transcriptome and metabolome combined KEGG analysis of Z23 at D3 vs. CK group. (**c**) Venn diagram of co-annotated KEGG pathways that were significantly enriched in the metabolome and transcriptome with *p* values ˂ 0.05.

The shared KEGG pathways of the transcriptomes and metabolomes of J432 and Z23 at D3 vs. CK were determined, respectively (Figure 6a,b), which resulted in 9 pathways (yellow cluster) and 13 pathways (blue cluster) (Figure 6c). Eight pathways were shared by the two clusters, which were mainly related to amino acid metabolism. There were five pathways unique to Z23 in the blue cluster, including the arginine and proline metabolism, Butanoate metabolism, TCA cycle, OXPHOS, and purine metabolism. Among them, the TCA cycle and OXPHOS are important components of respiratory metabolism.

**Figure 7:**
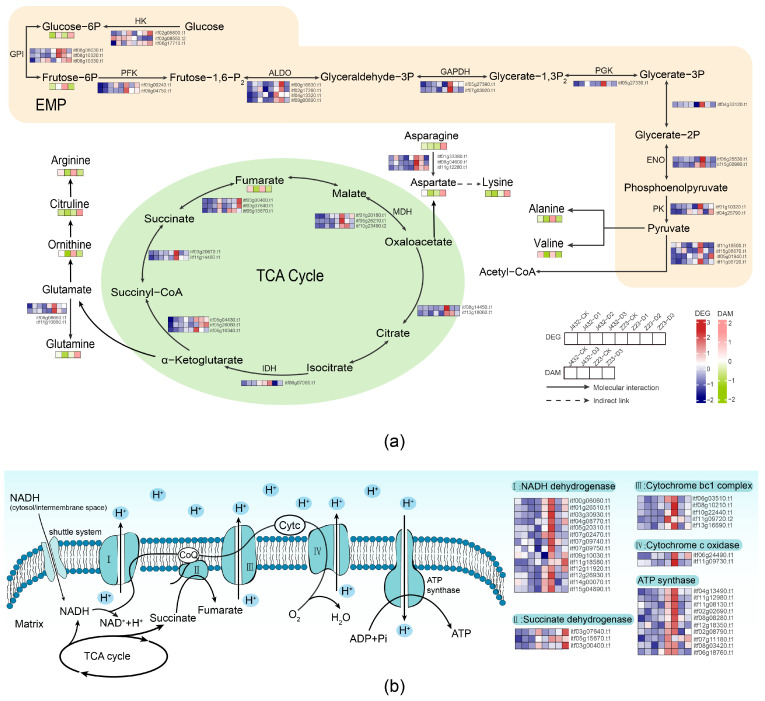
Effect of drought stress on amino acid metabolism and respiration metabolism. (**a**) The profiles of DEGs and DAMs in EMP, TCA cycle, and amino acid metabolism in response to drought. (**b**) The profiles of DEGs in OXPHOS in response to drought. Heatmaps colored in navy and firebrick indicate gene expression. Heatmaps colored in olive and pink indicate metabolite accumulation. HK, hexokinase; GPI, glucose-6-phosphate isomerase; PFK, phosphofructokinase; ALDO, fructose-bisphosphate aldolase; GAPDH, glyceraldehyde 3-phosphate dehydrogenase; PGK, phosphoglycerate kinase; ENO, enolase; PK, pyruvate kinase; IDH, isocitrate dehydrogenase; MDH, malate dehydrogenase.

To further analyze the respiratory metabolism, the content of metabolites and gene expression levels were mapped to the respiration metabolism’s KEGG pathways, including EPM, TCA cycle, and OXPHOS (Figure 7). We found that sixty-nine DEGs and three DAMs contributed to the high drought tolerance of Z23 in the EMP, TCA cycle, and OXPHOS pathways. Additionally, a total of nine amino acids were involved in the respiratory pathway by providing substrates (Tables S6 and S7).

## 3. Discussion

In this study, the drought-sensitive sweet potato cultivar J432 and drought-tolerant cultivar Z23 were treated under drought stress and their transcriptome and widely targeted metabolome data were analyzed. The results showed that drought stress induced the rapid realization of signal transduction, which, in turn, regulates the expression of TFs such as MYB, bHLH, and ERF. These TFs further activate the expression of downstream genes, which leads to drought responses including an enhanced EMP, enhanced TCA cycle, and enhanced OXPHOS metabolism intensity. They also increased antioxidant system levels, thereby a conferring higher drought tolerance on Z23 than J432. Therefore, the mechanism of drought tolerance in Z23 was elucidated from aspects of the respiratory metabolism, antioxidant system, and TFs.

### 3.1. Effects of Drought Stress on Respiration Metabolism

Drought and other stress conditions seriously interfere with the respiratory metabolism, affecting the release, transfer, storage, and utilization of plant energy, and thereby inhibiting plant growth and development. In this study, the results of WGCNA and combined transcriptome–metabolome analysis demonstrated that respiratory metabolism played an important role in the high drought tolerance of Z23.

Amino acids are important nitrogen sources in plants. Some amino acids can participate in energy production during drought by providing substrates to the TCA cycle and other respiratory pathways [32]. Plants with a higher amino acid content can better resist adversity stress [33]. In this study, nine amino acids contributed to the high drought tolerance of Z23. Compared with J432, the contents of aspartic acid, asparagine, lysine, glutamine and citrulline in Z23 significantly increased under drought treatment (Figure 7a). Although the contents of alanine, valine, ornithine, and arginine in both J432 and Z23 decreased under drought stress, their contents in the D3 treatment were still higher in Z23 than those in J432. The higher contents of these amino acids in Z23 can provide more fuel for mitochondrial respiration to produce energy and carbon intermediates to maintain the plant’s energy supply levels.

Plants can obtain a large amount of energy through the TCA cycle, EMP, and OXPHOS. In the EMP pathway, glucose is converted into glucose-6P by hexokinase (HK), which is then transformed into fructose-6P. Fructose-6P is further converted into fructose-1,6-P by phosphofructokinase (PFK), and then converted into phosphoenolpyruvate through a multi-step reaction. Phosphoenolpyruvate is then converted into pyruvate by pyruvate kinase (PK). HK, PFK, and PK serve as three unidirectional reaction points and are considered three rate-limiting steps in EMP. In this study, under drought stress, the expression levels of the genes encoding to HK, PFK, and PK in Z23 were increased and higher than those in J432, indicating that drought stress enhanced EMP metabolism and that the metabolic level of Z23 was greater than that of J432. The pyruvate generated in the EMP was transferred to the mitochondria and then converted into acetyl-CoA, which participates in the TCA cycle. Under drought stress, the expression of the genes encoding to most enzymes in the TCA cycle of J432 and Z23 showed a trend of first increasing and then decreasing. Additionally, compared with CK, the contents of fumarate and cis-aconitate in the TCA cycle of both J432 and Z23 were lower in D3. This finding indicates that, under severe drought stress, the level of TCA metabolism decreased. However, in Z23, TCA metabolism levels were still higher than in J432. Most of the energy produced by the EMP pathway and TCA cycle is stored in reducing coenzymes, but these coenzymes need to undergo OXPHOS to synthesize ATP, which is the universal energy currency of cells. OXPHOS is composed of inner mitochondrial membrane-embedded protein complexes I-IV and ATP synthase [34]. In this study, most of the genes encoding to complexes I-IV and ATP synthase in Z23 showed the highest expression levels in D1 stage and decreased with increasing drought severity (Figure 7b). By contrast, the expression levels of these genes in J432 did not show significant changes and were significantly lower than those in Z23. In general, the respiratory metabolism ability of Z23 was significantly enhanced under mild to moderate drought stress, but it was inhibited under severe drought stress. Compared with J432, Z23 has a stronger respiratory metabolism ability, which can better maintain the energy supply level under drought stress.

### 3.2. Effects of Drought Stress on Antioxidant System

The low concentrations of ROS produced by normal metabolic processes will not cause damage to plants [35], but drought stress can cause the production and accumulation of ROS. Excessive ROS will result in enzyme inhibition, DNA damage and biofilm peroxidation, which can lead to plant cell death in severe cases [36]. Antioxidant systems (both enzymatic and non-enzymatic) help plant cells remove excess ROS and improve plant stress tolerance. In this study, under drought stress, the POD and SOD activities of Z23 were higher than those of J432. In addition, KEGG enrichment analysis results showed that DEGs were enriched in ascorbate and aldarate metabolism, flavonoid biosynthesis, and anthocyanin biosynthesis, and the DAMs were enriched in the glutathione metabolism. Furthermore, DAMs classification results showed that Z23 had a higher content of flavonoids. These results suggest that the antioxidant capacity of Z23 may be stronger than that of J432.

**Figure 8:**
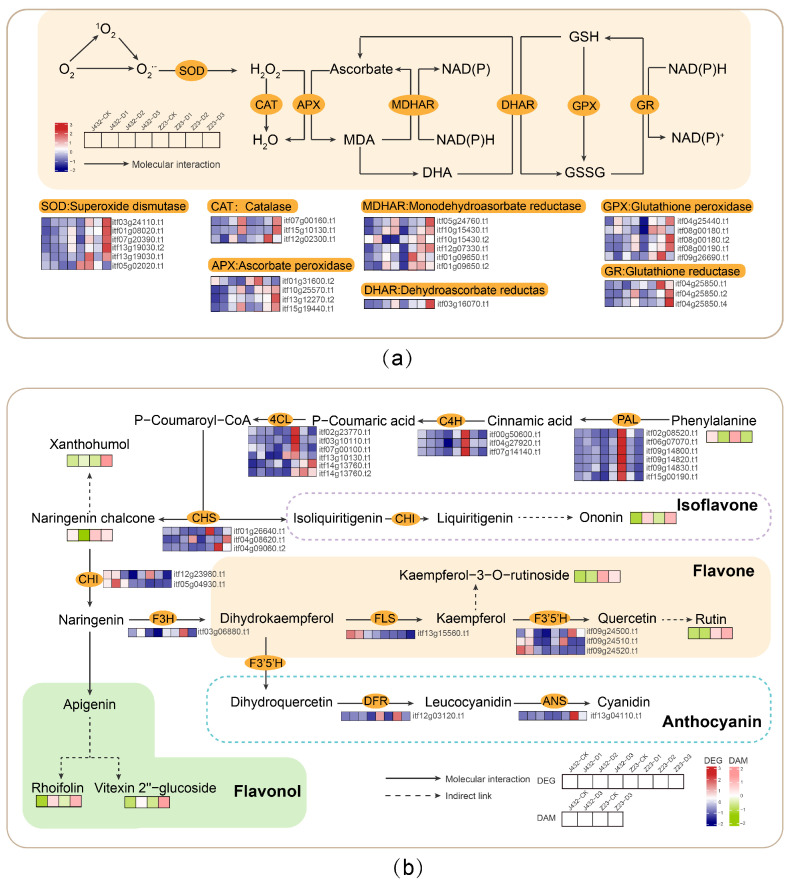
Effects of drought stress on antioxidant systems in sweet potato. (**a**) Effects of drought stress on enzymatic system. (**b**) Effects of drought stress on the non-enzymatic system. Heatmaps colored in navy and firebrick indicate gene expression. Heatmaps colored in olive and pink indicate metabolite accumulation. MDA, monodehydroascorbate; DHA, dehydroascorbate; NADP+/NADPH, nicotinamide adenine dinucleotide phosphate; NAD+/NADH, nicotinamide adenine dinucleotide; GSH, reduced glutathione; GSSG, oxidized glutathione; PAL, phenylalanine ammonia-lyase; C4H, cinnamate-4-hydroxylase; 4CL, 4-coumaroyl-CoA ligase; CHS, chalcone synthase; CHI, chalcone isomerase; F3H, flavanone-3-hydroxylase; FLS, flavonol synthase; F3′5′H, flavonoid 3′,5′-hydroxylase; DFR, bifunctional dihydroflavonol 4-reductase; ANS, anthocyanidin synthase.

The enzymatic antioxidant system mainly includes SOD, POD, CAT, ascorbate peroxidase (APX), dehydroascorbate reductase (DHAR), monodehydroascorbate reductase (MDHAR), glutathione peroxidase (GPX), glutathione reductase (GR), and other protective enzymes. In this study, the POD and SOD activities of the two cultivars were increased under drought stress, and both activities were significantly higher in Z23 than in J432 at each stage, from D1 to D3 (Figure 1e,f). Transcriptome data showed that the genes encoding six SOD, three CAT, four APX, six MDHAR, one DHAR, five GPX and three GR enzymes were significantly induced by drought stress in J432 and Z23; however, their expression levels were much higher in Z23 (Figure 8a, Table S8). These results indicate that the enhanced levels of antioxidant activity contribute to the higher drought tolerance of Z23.

The non-enzymatic antioxidant system mainly includes some organic compounds with a small relative molecular mass, such as ascorbic acid, glutathione, flavonoids, and non-protein amino acids. Flavonoids play a variety of roles in plants and are an important class of ROS-scavenging substances [37]. Studies have shown that flavonoids can help alleviate oxidative stress, reduce water loss in plant tissues, and enhance plant drought tolerance [38]. At present, the flavonoid synthesis pathways in plants have been well characterized [39]. In this study, the genes encoding to phenylalanine ammonia lyase (PAL), cinnamic acid-4-hydroxylase (C4H), and 4-coumarate coenzyme A ligase (4CL) in the flavonoid biosynthesis pathway were more highly expressed at the D1 stage in Z23 compared with J432, indicating the greater synthesis of flavonoids from phenylalanine in Z23 (Figure 8b, Tables S8 and S9). There was a greater consumption of phenylalanine—the initial substance in the flavonoid synthesis pathway—in Z23 from CK to D3. The contents of flavonoids such as rutin, xanthohumol, rhoifolin, ononin, and vitexin2″-glucoside increased in both J432 and Z23 under drought treatment, although they were significantly higher in Z23 than in J432. In this study, during the D3 period, the rutin content in Z23 was 43 times that of J432. Previous studies have shown that drought and heat stress appeared to induce the accumulation of rutin [40]. Rutin plays an important role in improving plant tolerance to abiotic stress [41]. Therefore, under drought stress, Z23 can synthesize more flavonoids to improve its drought tolerance, and rutin may play an important role in the high drought resistance of Z23.

### 3.3. Effects of Drought Stress on Plant Hormone Signal Transduction

The response of plants to drought stress involves a series of complex processes ranging from single-gene to whole-organism levels. The ability of plants to sense stimuli, and produce and transduce signals, is closely related to their drought tolerance. According to KEGG annotation, important differentially expressed TFs were mainly related to plant hormone signal transduction (Figure S2b). Combined with metabolomics data, it was found that ABA, JA, and GA signal transduction may play an important role in the high drought resistance of Z23.

**Figure 9:**
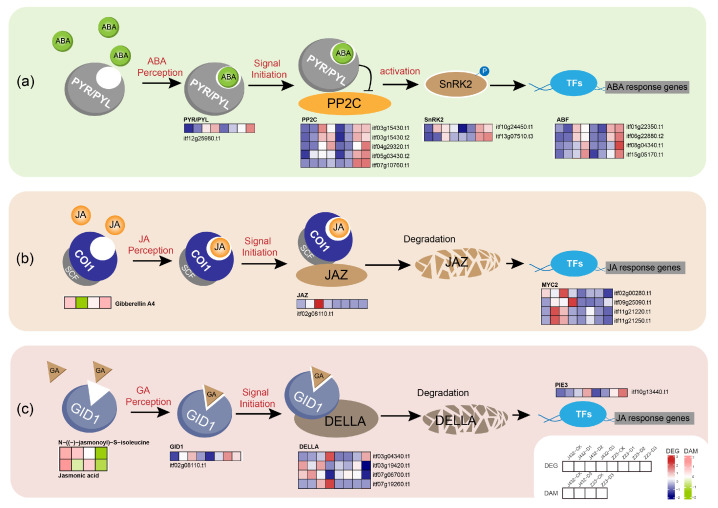
Effects of drought stress on plant hormone signal transduction. (**a**) Effects of drought stress on ABA signal transduction. (**b**) Effects of drought stress on JA signal transduction. (**c**) Effects of drought stress on GA signal transduction. Heatmaps colored in navy and firebrick indicate gene expression. Heatmaps colored in olive and pink indicate metabolite accumulation. ABA, abscisic acid; PYR/PYL, abscisic acid receptor PYR/PYL family; PP2C, protein phosphatase 2C; SnRK2, sucrose non-fermenting 1-related protein kinase 2; ABF, ABA-responsive element binding factor; JA, jasmonic acid; COI1, coronatine-insensitive protein 1; SCF, skp1-cullin-F-box protein; JAZ, jasmonate ZIM domain-containing protein; MYC2, basic helix–loop–helix; GA, gibberellinic acid; GID1, GA-INSENSITIVE DWARF1; DELLA, DELLA protein; PIE3, phytochrome-interacting factor 3.

The plant hormone ABA is a key signal regulator in many vital processes; approximately 10% of the signal genes in Arabidopsis thaliana are regulated by ABA [42]. When plants are under drought stress, ABA enters cells through ABC transporters, binds to PYR/PYL-type ABA receptor and changes their conformation so that they can interact with protein phosphatase 2C (PP2C), thereby inhibiting PP2C protein phosphatase activity and allowing for sucrose non-fermenting 1-related protein kinase 2 (SnRK2) to be phosphorylated and activated [43]. Subsequently, downstream TFs such as ABF are activated, and a series of reactions, such as stomatal closure and antioxidant synthesis, are initiated. Under drought stress, one PYR/PYL receptor-related gene, five PP2C genes, two SnRK2-encoding genes and four ABF TFs showed a positive regulation of drought stress in Z23 (Figure 9a, Table S10). The higher ABA signal transfer efficiency might thus contribute to the greater drought tolerance of Z23.

While JA-mediated defense responses aid in plants’ adaptation to various biotic and abiotic stresses, prolonged and uncontrolled responses can have adverse effects [44]. In this study, it was detected that, in Z23, the contents of JA and an active form of JA (N-((-)-jasmonoyl)-S-isoleucine) significantly decreased under severe drought compared to the CK, and were notably lower than J432. The proper termination of JA-mediated defense responses may be one of the reasons for the high drought resistance of Z23. When plants are subjected to stress, active JA is recognized by the SCF (COI1) ubiquitin ligase that targets the repressor protein JA zim domain (JAZ) for degradation in the 26S. The removal of JAZ releases the activity of the core transcription factor MYC2, thereby activating the expression of JA response genes to produce a defense response [45]. In this study, one JAZ repressor gene and four MYC2 TFs were detected, and under drought stress; their expression levels in Z23 and J432 showed a rapid increase followed by a decline, with lower expression levels in Z23 compared to J432 (Figure 9b, Table S10). This expression pattern may be related to a self-regulatory feedback mechanism to prevent excessive defense in plants. The timely inhibition and termination of the JA signaling pathway may be beneficial for the growth and development of sweet potato under drought stress.

Under drought stress, the GA content increases in Z23 while decreasing in J432, potentially contributing to Z23’s higher drought resistance. In the GA signal transduction model, the binding of GA to GID1 (GA-INSENSITIVE DWARF1) promotes the interaction between GID1 and the DELLA repressor, leading to DELLA degradation through the ubiquitin–proteasome pathway, which, in turn, causes a GA response [46]. In this study, the expression levels of one GID1 gene and a target gene of DELLA, PIF3, were upregulated in Z23 under drought stress compared to J432. Additionally, the expression levels of four DELLA genes were higher in J432, favoring GA signal transduction (Figure 9c, Table S10). Furthermore, previous studies have demonstrated direct interactions between DELLA and key components of almost all hormone pathways. For example, DELLA can interact with JAZ and MYC2 to regulate cross-talk between GA and JA. The complex plant hormone signal transduction network may play a crucial regulatory role in the rapid response of sweet potato to drought stress.

### 3.4. Effects of Drought Stress on Transcription Factors

TFs play a vital role in regulating plant drought tolerance by activating or inhibiting gene expression, regulating plant growth and development, environmental stress responses, and the biosynthesis of secondary metabolites [47]. After being subjected to environmental stress, plants activate TFs through a series of stress signal transductions, which bind to the corresponding cis-elements in the downstream target-gene-promoter region, regulating target gene expression in response to stress signals. Previous studies have shown that many TF families have been proven to be useful in improving plant drought tolerance in sweet potato [48,49,50,51]. According to our data, among 158 TFs, the ERF, bHLH, GRAS, MYB-related, and WRKY members were relatively large TF families in response to drought. The ERF family genes account for 10.75%. We identified IbRAP2–3 (itf04g20580.t3), a gene encoding the ERF transcription factor. RNA-seq and qRT-PCR results showed that its expression was significantly induced by drought stress, and the expression levels in Z23 at D1, D2, and D3 were 8, 3, and 3.4 times those in J432 (Figure 4). Homologous gene AT3G16770 of IbRAP2–3 in Arabidopsis thaliana is part of the ET signaling pathway and is predicted to act downstream of EIN2 and CTR1, but not under EIN3 [52]. Therefore, IbRAP2–3 may regulate drought resistance in sweet potato through the ET signaling pathway. The bHLH family is one of the largest transcription factor families in plants, accounting for 8.23% in this study. QRT-PCR results showed that the expression of IbFBH1 (itf04g21760.t1) in Z23 at D2 and D3 was 2.4 and 1.9 times that in J432 (Figure 4). Its homologous gene in Arabidopsis thaliana is involved in the CFL1-mediated regulation of cuticle development [53]. The cuticle, composed of cutin and waxes, can protect plants against biotic and abiotic stresses. The drought-resistance mechanism of IbFBH1 in sweet potato may also be related to cuticle development. The MYB-related gene family accounted for 6.96% of the differentially expressed TFs, and qRT-PCR results showed that, under drought stress, the expression of IbMYB48 (itf04g30040.t2) in Z23 is 3–8 times that of J432 (Figure 4). Previous research has shown that the ABA biosynthetic pathway, JA biosynthesis and signaling pathway, and ROS-scavenging system are activated by the overexpression of IbMYB48, thus improving the drought stress tolerance of transgenic Arabidopsis [54]. This further confirms the reliability of this study’s results. BZIP is a widely distributed class of transcriptional regulatory factors in plants. Here, we found that one bZIP transcription factor, IbZIP58 (itf13g22080.t1), showed a significant difference in expression between two sweet potato varieties, with the expression in Z23 being 20 times higher than in J432 at the D2 stage (Figure 4). This may contribute to the higher drought tolerance of Z23. These genes have great potential to improve the drought tolerance of sweet potato through genetic manipulation in future studies.

## 4. Materials and Methods

### 4.1. Plant Materials and Treatments

Sweet potato cultivar J432 and Z23 were grown in transplanting boxes for continuous drought stress treatment in a greenhouse. Four boxes were used, with three 25 cm long cuttings of field-grown J432 and Z23 cultured in each box with the same amount of soil mix (3: 1, nutrient soil: vermiculite). Each box was watered an equal amount once for plant recovery and then submitted to natural drought stress. As described by Zhou et al. [55], RSMC is an effective index to describe the degree of drought. In this study, four treatments were set, namely, CK, D1, D2, and D3. The four transplanting boxes were weighed between 16:00 and 17:00 every day, and water was supplemented to maintain the four corresponding RSMC levels. When the D3 box reached its target RSMC of 6%, after about three weeks, all treatments were concluded and the main vine length of each plant was measured.

### 4.2. Morphological and Physiological Assessment under Drought Stress

Once the RSMC reached the target value for each treatment, one leaf was taken from each plant at the same position for LRWC measurement. The fresh weight (FW) of the leaves was recorded before they were soaked in distilled water at 4 °C for 24 h. The saturated fresh weight (TW) was subsequently determined, and the leaves were dried in an oven at 75 °C for 24 h. The dry weight (DW) was measured and the LRWC was calculated as follows:

$$LRWC\left(\%\right)=\frac{FW-DW}{TW-DW}\times 100$$

The proline content and the activities of SOD and POD in leaves were measured using assay kits (Sangon Biotech, Shanghai, China). Three biological replicates were used for each treatment. * and ** indicate significant differences (at *p* < 0.05 and *p* < 0.01, respectively) between J432 and Z23 at each drought level based on Student’s *t*-test.

### 4.3. Sampling for Transcriptome and Metabolome Analyses

Once the RSMC reached the target value for each treatment, one leaf was taken from each plant at the same position and quickly frozen in liquid nitrogen before storage at −80 °C. Three biological replicates were used for each treatment. A total of 24 leaf samples of two cultivars and four treatments (CK, D1, D2, D3) were then submitted for transcriptome sequencing. Additionally, 12 sample leaves of two treatments (CK and D3) with the most significant morphological and physiological differences between the two cultivars were used for widely targeted metabolome analysis.

### 4.4. RNA Extraction and Transcriptome Sequencing

Total RNA was extracted using Trizol reagent (Invitrogen, Carlsbad, CA, USA) according to the manufacturer’s protocol. Eukaryotic mRNA was enriched by Oligo (dT) beads. Then, the enriched mRNA was fragmented into short fragments using a fragmentation buffer and reverse-transcribed into cDNA using NEBNext Ultra RNA Library Prep Kit for Illumina (NEB #7530, New England Biolabs, Ipswich, MA, USA). The resulting cDNA library was sequenced using an Illumina Novaseq 6000 (Illumina, San Diego, CA, USA). The clean reads obtained by deleting the sequencing adaptors and low-quality bases were mapped to the reference genome (http://sweetpotato.uga.edu/index.shtml, accessed on 5 October 2022) of sweet potato wild relative *Ipomoea trifida*. Transcript abundances were quantified and normalized based on FPKM. PCA was performed on variance-stabilized transformed values and regularized log values of read counts, respectively. DEGs were defined with an adjusted *p*-value ≤ 0.05 and |log2 (fold change)| > 1 using the DEseq2 (v1.24.0). Finally, DEGs were described using GO and KEGG analyses.

The co-expression network was constructed using the WGCNA (v1.47) package in R. After filtering genes, we calculated the correlation coefficients between module eigengene values and samples or sample traits to identify biologically significant modules. Genes were clustered into 18 correlated modules.

### 4.5. Metabolite Extraction and LC-MS/MS Analysis

Widely targeted metabolome analysis was used to analyze the metabolites of 12 samples. QC samples were used to control the quality of the experiment. The LC-MS/MS analyses were performed using ultra-high-performance liquid chromatography (EXION LC System, SCIEX, Framingham, MA, USA) coupled with an SCIEX 6500 QTrap and Triple Quadrupole Mass Spectrometer. All mass spectrometry data acquisition and quantitative analyses of target compounds were performed with SCIEX Analyst Work Station Software (version 1.6.3). DAMs were identified by using a threshold value of *p* ≤ 0.05 and variable importance in project (VIP) ≥ 1. The functions of DAMs were further annotated using the KEGG compound database.

### 4.6. Quantitative Real-Time PCR of Candidate Genes

qRT-PCR of eight genes that potentially contribute to drought tolerance was performed on the CFX96 Real-Time PCR Detection System (Bio-Rad, Hercules, CA, USA). *IbActin* (GenBank Accession Number is AY905538) was used as an internal control. All primers that were used are listed in Table S11. The relative gene expression levels were calculated using the 2^−ΔΔCT^ method. Values are the means of three replicates. Data are presented as means ± SD (*n* =3). * and ** indicate significant differences compared with CK at *p* < 0.05 and <0.01, respectively, based on Student’s *t*-test.

## 5. Conclusions

In this study, the drought-sensitive sweet potato cultivar J432 and drought-tolerant cultivar Z23 were treated under drought stress and their transcriptome and widely targeted metabolome data were analyzed. The results showed that drought stress resulted in the accumulation of ROS in Z23. ROS can be used as secondary messengers to induce the rapid realization of plant hormone signal transduction, which, in turn, regulates the expression of TFs such as MYB, bHLH, and ERF. These TFs further activate the expression of downstream genes, which leads to drought responses including enhanced EMP, an enhanced TCA cycle, and enhanced OXPHOS metabolism intensity, as well as increased antioxidant system levels, thereby conferring higher drought tolerance to Z23 compared to J432 (Figure S4). Therefore, the mechanism of drought tolerance in Z23 was elucidated from aspects of the respiratory metabolism, antioxidant system, and TFs.

## Data Availability

The transcriptome datasets in this study were stored in NCBI under the accession number PRJNA1039563.

## Supplementary Materials

The following supporting information can be downloaded at: https://www.mdpi.com/article/10.3390/plants13030351/s1, Figure S1: Transcriptome supplementary analysis. (a) PCA of 24 samples. (b) The numbers of DEGs in the different comparison groups. (c) GO enrichment analysis of 93 common DEGs in Figure 2a. (d) GO enrichment analysis of 112 common DEGs in Figure 2c; Figure S2: Transcription factor analysis. (a) One hundred and fifty-eight TFs classifications. (b) KEGG pathway analysis of 158 TFs; Figure S3: Gene expression profile for ‘lightcyan1’ and ‘skyblue’ modules in different samples. The heatmap above shows the expression profiles of all co-expressed genes; Figure S4: The putative mechanism of the response of sweet potato under drought stress; Table S1: Throughput and quality summary of RNA-sequences; Table S2: FPKM values of 93 upregulated DEGs and 112 downregulated DEGs in Z23 compared with J432 at the D1, D2, and D3 levels; Table S3: FPKM values of 158 transcription factors; Table S4: Details of 793 metabolites detected in the metabolome; Table S5: Details of 61 DAMs from clusters 1 and 6; Table S6: FPKM values of DEGs related to amino acid metabolism and respiration metabolism; Table S7: Details of DAMs related to amino acid metabolism and respiration metabolism; Table S8: FPKM values of DEGs related to antioxidant systems; Table S9: Details of DAMs related to antioxidant systems; Table S10: FPKM values of DEGs related to plant hormone signal transduction. Table S11: Primer sequences used for qRT-PCR analysis in this article.
