# Supplementary material for: Transcriptome and Metabolome Analyses Reflect the Molecular Mechanism of Drought Tolerance in Sweet Potato
Source: Plants (Basel). 2024 Jan 24;13(3):351. doi: 10.3390/plants13030351 (PMC10857618; doi:10.3390/plants13030351)
Supplement: Supplementary file 1 [file plants-13-00351-s001.zip › Supplementary Figures.pdf]

Supplementary Materials

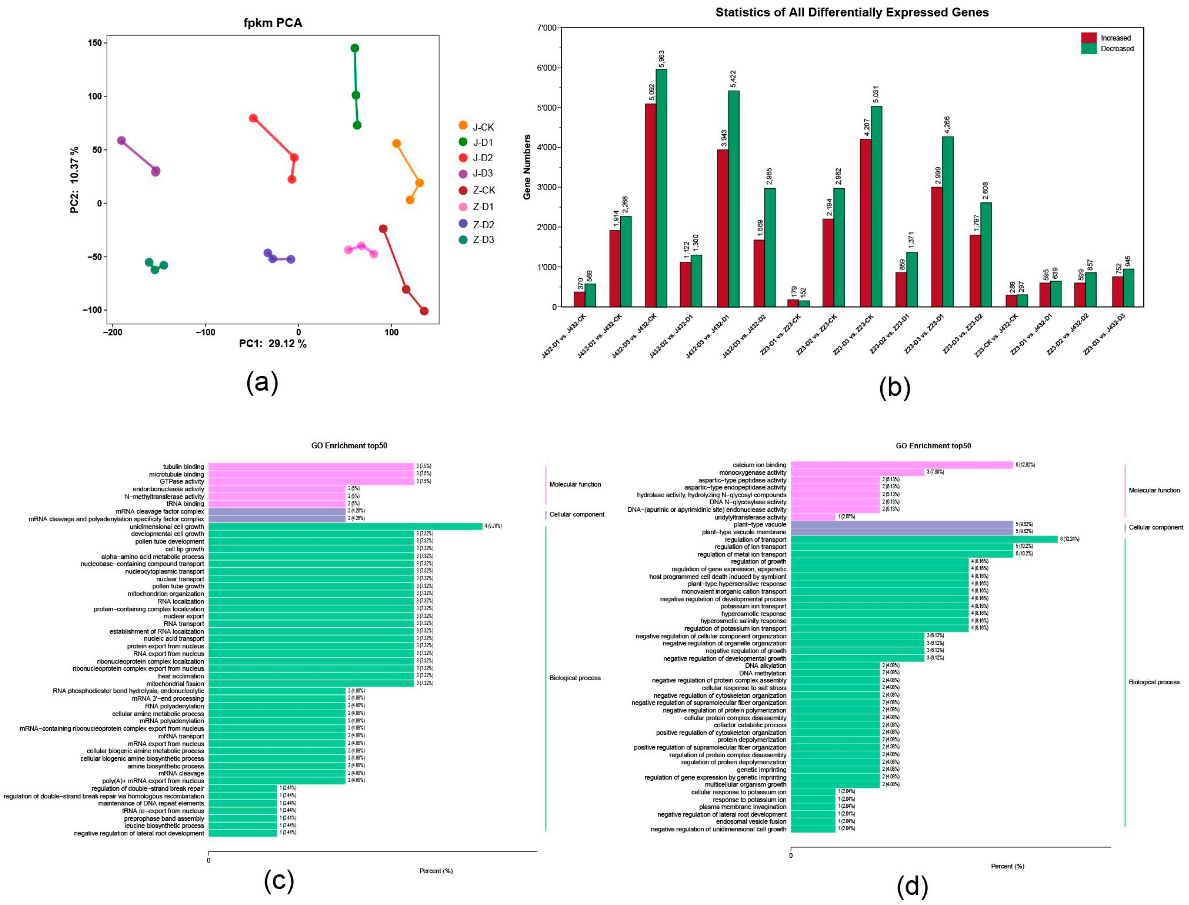

Figure S1: Transcriptome supplementary analysis. (a) PCA of 24 samples. (b) The numbers of DEGs in the different comparison groups. (c) GO enrichment analysis of 93 common DEGs in figure 2a. (d) GO enrichment analysis of 112 common DEGs in figure 2c.

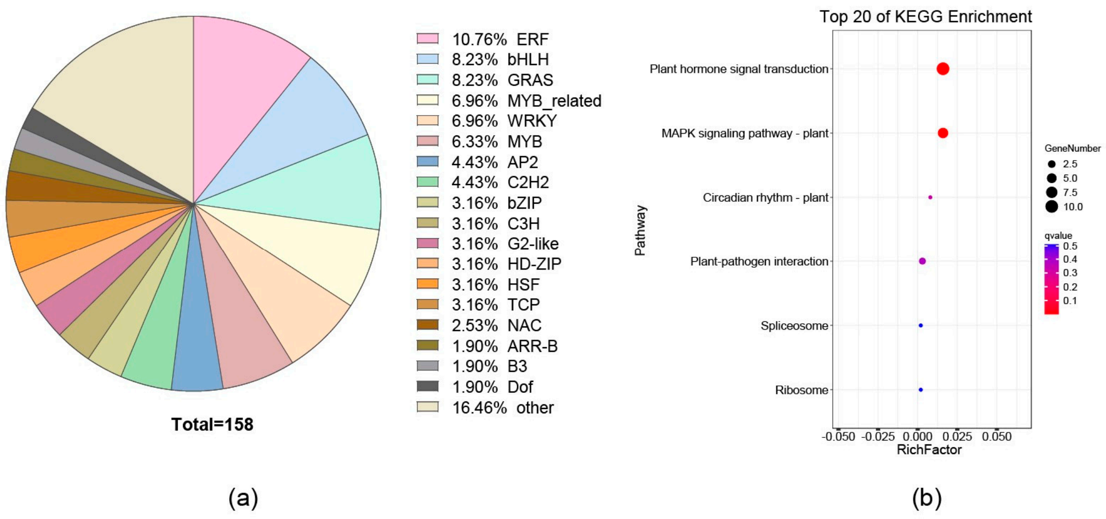

Figure S2: Transcription factor analysis. (a) One hundred and fifty-eight TFs classifications. (b) KEGG pathway analysis of 158 TFs.

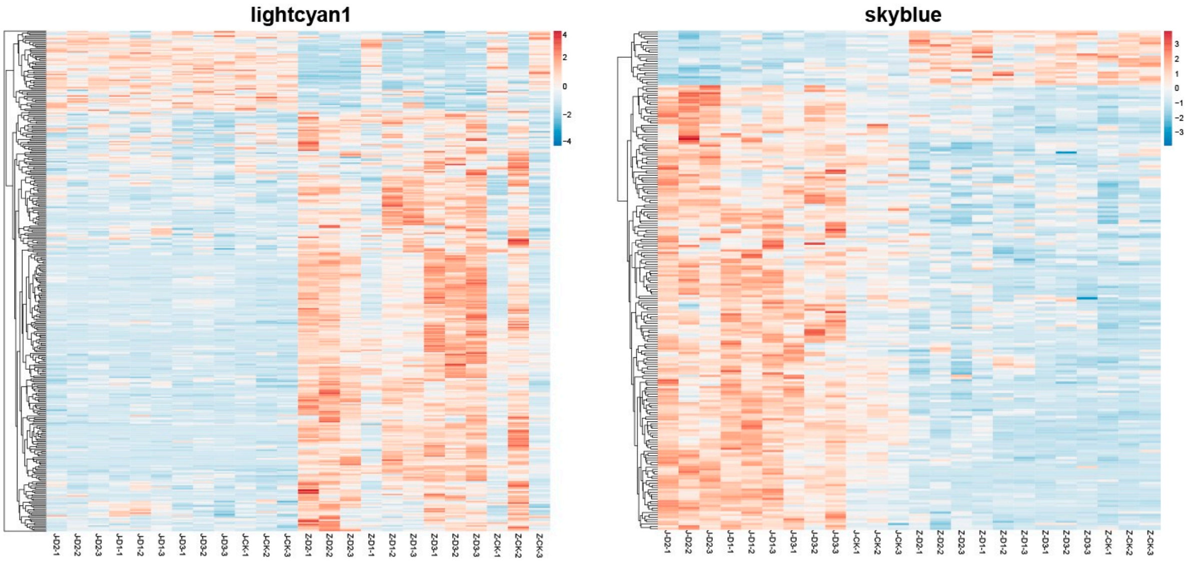

Figure S3: Gene expression profile for 'lightcyan1' and 'skyblue' modules in different samples. The heatmap above shows the expression profiles of all co-expressed genes.

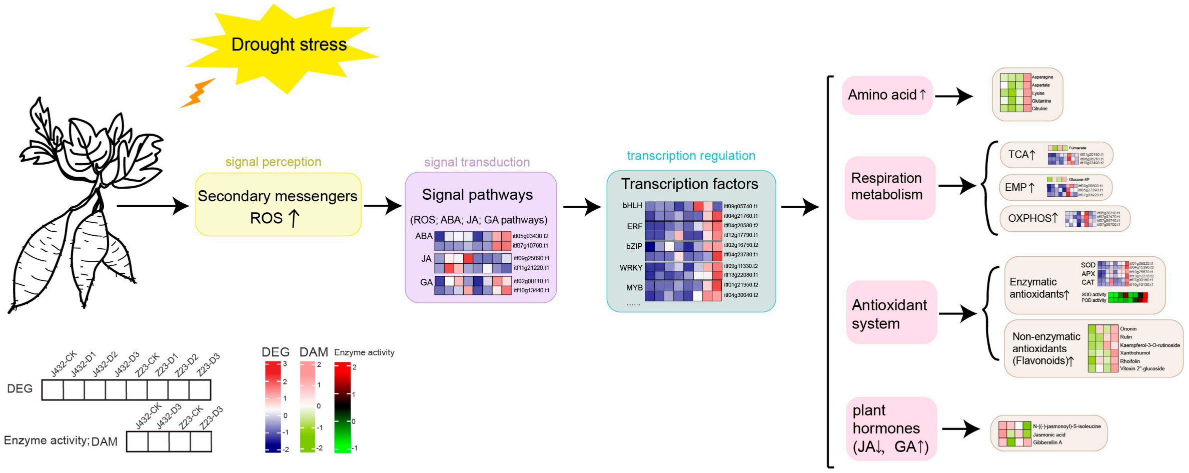

Figure S4: The putative mechanism of the response of sweet potato under drought stress.
